# Vertical suppression of the EGFR pathway prevents onset of resistance in colorectal cancers

**DOI:** 10.1038/ncomms9305

**Published:** 2015-09-22

**Authors:** Sandra Misale, Ivana Bozic, Jingshan Tong, Ashley Peraza-Penton, Alice Lallo, Federica Baldi, Kevin H. Lin, Mauro Truini, Livio Trusolino, Andrea Bertotti, Federica Di Nicolantonio, Martin A. Nowak, Lin Zhang, Kris C. Wood, Alberto Bardelli

**Affiliations:** 1 Candiolo Cancer Institute — Fondazione Piemontese per l'Oncologia (FPO), Istituto di Ricovero e Cura a Carattere Scientifico (IRCCS), Candiolo, Torino, 10060, Italy; 2 FIRC Institute of Molecular Oncology (IFOM), Milano 20139, Italy; 3 Program for Evolutionary Dynamics, Harvard University, Cambridge, Massachusetts 02138, USA; 4 Department of Mathematics, Harvard University, Cambridge, Massachusetts 02138, USA; 5 University of Pittsburgh Cancer Institute, Pittsburgh, Pennsylvania 15213, USA; 6 Department of Pharmacology and Chemical Biology, University of Pittsburgh School of Medicine, Pittsburgh, Pennsylvania 15213, USA; 7 Department of Pharmacology and Cancer Biology, Duke University, Durham, North Carolina 27710, USA; 8 University of Torino, Department of Oncology, SP 142, Km 3.95, 10060 Candiolo, Torino, Italy; 9 Division of Pathology, Niguarda Cancer Center, Ospedale Niguarda Ca' Granda, 20162 Milano, Italy; 10 Department of Organismic and Evolutionary Biology, Harvard University, Cambridge, Massachusetts 02138, USA

## Abstract

Molecular targeted drugs are clinically effective anti-cancer therapies. However, tumours treated with single agents usually develop resistance. Here we use colorectal cancer (CRC) as a model to study how the acquisition of resistance to EGFR-targeted therapies can be restrained. Pathway-oriented genetic screens reveal that CRC cells escape from EGFR blockade by downstream activation of RAS-MEK signalling. Following treatment of CRC cells with anti-EGFR, anti-MEK or the combination of the two drugs, we find that EGFR blockade alone triggers acquired resistance in weeks, while combinatorial treatment does not induce resistance. In patient-derived xenografts, EGFR-MEK combination prevents the development of resistance. We employ mathematical modelling to provide a quantitative understanding of the dynamics of response and resistance to these single and combination therapies. Mechanistically, we find that the EGFR-MEK Combo blockade triggers Bcl-2 and Mcl-1 downregulation and initiates apoptosis. These results provide the rationale for clinical trials aimed at preventing rather than intercepting resistance.

## Abstract (web summary)

Cancer patients often respond well to primary treatment but then develop resistance. Here, Misale *et al*. show that dual treatment with EGFR and MEK inhibitors block resistance in mice containing patient-derived xenografts and provide a mathematical model that describes the temporal development of resistant tumour clones.

## Graphical abstract

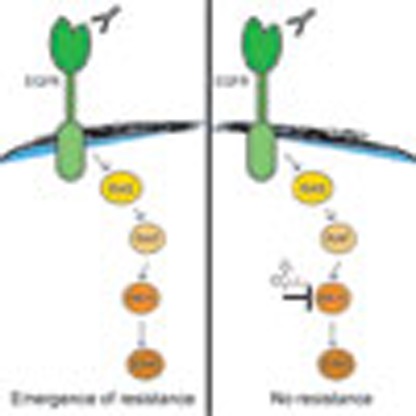

The evidence that cancer is a malady of genes has revolutionized diagnosis and treatment of this disease^1^,^2^. A central paradigm of modern oncology is that tumour-specific molecular alterations (such as *HER2* amplification or *EGFR* and *BRAF* mutations) underlie ‘functional' dependencies that can be therapeutically exploited^3^. Remarkable results have been obtained by applying this paradigm in the clinic and several ‘targeted' drugs were approved to treat, among others, melanomas, lung and colorectal cancers (CRCs). When ‘targeted' agents were employed in the clinic it quickly became apparent that patients treated with single agents almost always develop resistance within months after initiating therapy^4^,^5^,^6^,^7^,^8^,^9^.

A typical observation is that after an initial regression all the metastatic lesions reappear, virtually simultaneously^10^. How can we overcome the near-certainty of disease recurrence following treatment with targeted agents? If resistance is directly dependent on the number of cells that are treated, in principle, the best options would be to treat tumours when they are very small, before a sufficient number of mutant cells conferring resistance have accumulated. Unfortunately, this option is presently unfeasible as most tumours are discovered when cancerous lesions already contain billion of cells. Another feature, which limits the efficacy of targeted agents, is molecular heterogeneity. Solid tumours typically display high levels of genomic instability, which fuels the perpetual generation of molecular diversity. As a consequence, therapeutic challenges result in selection of sub-clonal cell populations, capable of growing under drug pressures^11^.

A possible approach to overcome the limitations of targeted agents is to use combinations rather than mono-therapies. This involves treating tumours with two or more agents that hit different targets, as this should reduce the chance of having cells resistant to both drugs^12^. Combination therapy is supported by the success of pharmacological combinations for systemic infectious diseases such as HIV^13^. In oncology, experimental evidence that combinations of targeted agents confer advantages over sequential treatments arise from melanoma patients treated concomitantly with anti-BRAF and anti-MEK drugs^14^.

In this work, we studied CRC as a model system to assess how the emergence of resistance to targeted therapies can be delayed or prevented. The anti-epidermal growth factor receptor (EGFR) antibodies cetuximab and panitumumab are used to treat metastatic CRCs lacking RAS pathway mutations^4^,^15^. Unfortunately, CRC patients who respond to EGFR antibodies almost invariably develop resistance within several months of initiating therapy^16^,^17^. Currently, there are no effective ways to control the onset of acquired resistance to EGFR blockade. We reasoned that it may be preferable to prevent the emergence of resistance rather than targeting tumour cells that had already acquired resistance. Functional genetic screening and pharmacological approaches demonstrated a strong dependency from EGFR-MEK pathway, which we exploited as a combinatorial treatment (EGFR plus MEK inhibition) on CRC cells and xenopatients. Our results demonstrate that EGFR-MEK double blockade limits the emergence of resistant clones preventing secondary resistance by inducing apoptosis more effectively then single agents alone. Mathematical modelling allowed quantitative understanding of the dynamics of response and resistance to mono and combination therapies.

## Results

### MAPK pathway activation drives resistance to EGFR blockade

**Figure 1:**
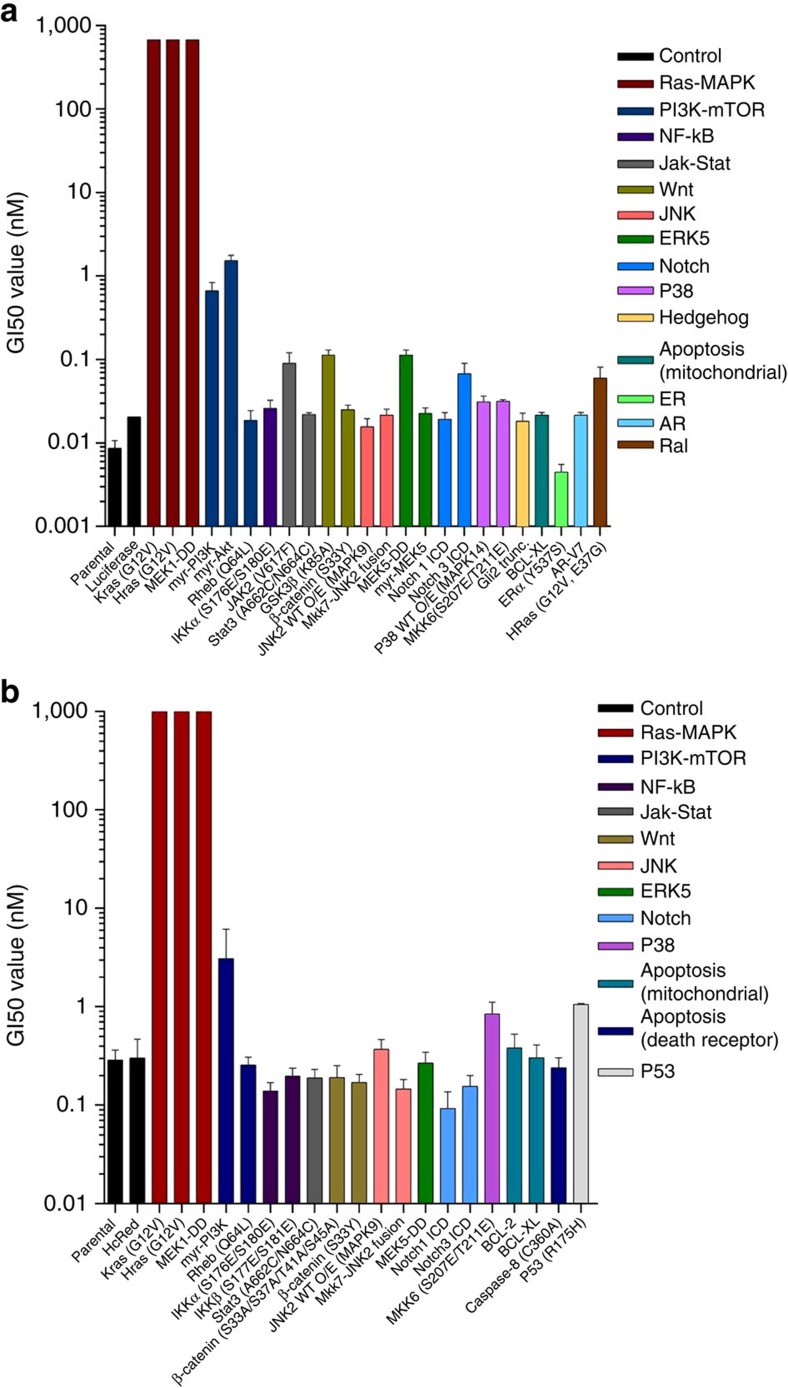
The RAS-MAPK pathway drives resistance to EGFR blockade in CRC cells. CCK81 (**a**) and DiFi (**b**) cells were infected with pathway specific cDNAs and treated with panitumumab. GI50 values are reported in logarithmic scale. Data are representative of three biological replicates and error bars represent s.d. Details on the constructs are listed in Supplementary Table 1.

To identify signalling pathways with the potential to confer resistance to anti-EGFR antibodies, we performed a functional genetic screen. Two CRC cell models (DiFi and CCK81) sensitive to EGFR blockade were infected with a library of 27 pathway-activating mutant cDNAs (Supplementary Table 1). For most signalling pathways, multiple Open Reading Frames (ORFs) were included that on ectopic expression, rendered the pathway constitutively active. Each construct was individually barcoded thus allowing sequence-based screen, and most constructs were functionally validated to ensure constitutive activation of their cognate pathways in cells^18^. Constitutive activation of RAS-MAPK signalling (but not of other pathways) was sufficient to confer resistance to EGFR blockade (Fig. 1a,b). Comparable results were obtained with two independent CRC cell lines (Supplementary Fig. 1).

### Dual blockade of EGFR and MEK prevents acquired resistance

If MAPK activation is necessary to sustain resistance to EGFR blockade, we reasoned that vertical suppression of EGFR signalling by concomitant EGFR-MEK targeting could delay or prevent the emergence of resistant clones. To experimentally verify the hypothesis we devised a cell-based test that parallels time to progression (TTP), a parameter commonly used in the clinic. In cancer patients, TTP is defined as the time from the start of treatment until disease worsens or spreads to other parts of the body because of tumour growth. The *in vitro* TTP assay defines the emergence of acquired resistance as the time at which a fixed number of cells start to grow in an exponential manner in the presence of drug(s). We found that in the presence of cetuximab, time to resistance depends on the initial cell input (Supplementary Fig. 2). An increased number of input cells correlated with a shorted TTP, consistent with the hypothesis that cells resistant to EGFR blockade are pre-existing^17^ (Supplementary Fig. 2).

**Figure 2:**
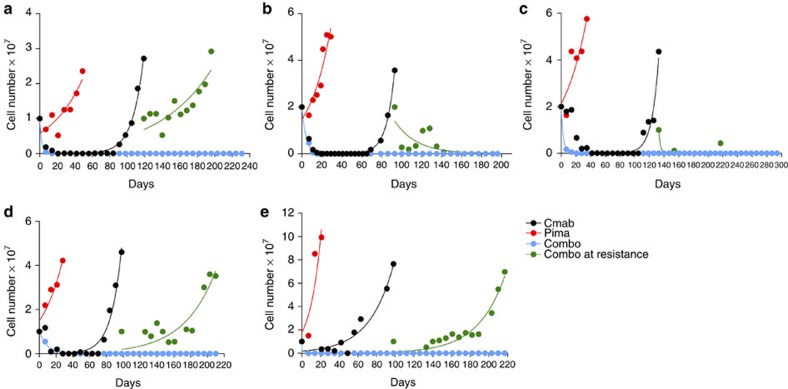
Concomitant blockade of EGFR and MEK halts the emergence of resistance in CRC cell lines. CCK81 (**a**), DiFi (**b**), C99 (**c**), NCIH508 (**d**) and HCA-46 (**e**). Cell lines were seeded at 20 million (CCK81 and C99) and 10 million (DiFi, NCIH508 and HCA-46) cell density and treated with cetuximab alone (340 nM), pimasertib alone (250 nM) and with the combination of the two drugs from day 0 or at the time of acquired resistance to cetuximab. Cells were detached and counted at least once a week as described in Methods section. A single biological replicate is represented for each cell model. Non-linear fit with exponential growth curve (Graphpad Prism) was applied to data points to show growth kinetics.

In DiFi and CCK81, the TTP assay shows that inhibition of MEK alone had modest or no impact. We further found that resistance to cetuximab occurs within 80–120 days after therapy initiation depending on the cell line. Similar results were obtained in multiple CRC cell models (Fig. 2).

We next asked what would happen if the EGFR-MEK combo treatment was applied to population of cells at the time they developed acquired resistance to cetuximab. Some of the cetuximab-resistant models displayed sensitivity to the EGFR-MEK therapy (CCK81 and C99) while others, after a short-lived growth inhibition, quickly evaded combinatorial treatment (Fig. 2).

Most notably, concomitant suppression of EGFR and MEK in treatment-naive cells had a remarkable effect, as we were unable to detect emergence of resistant clones up to 6 months after initiation of treatment (Fig. 2). Of note, we found that high dose mono-agents are not cytotoxic (Supplementary Fig. 3).

### Combinatorial therapy prevents resistance in CRC PDX

**Figure 3:**
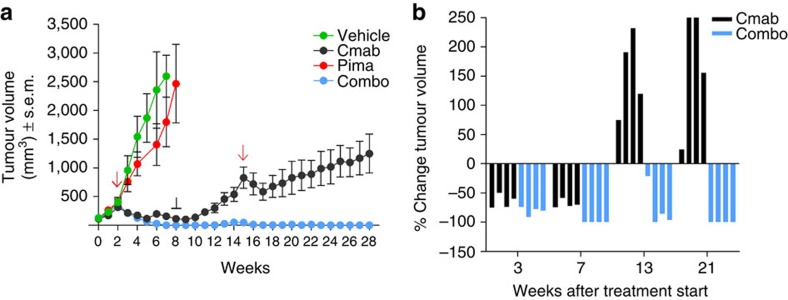
Vertical blockade of the EGFR pathway prevents the emergence of resistance in PDX. A cetuximab sensitive CRC PDX (xenopatient) was expanded to create four cohorts of four mice each. (**a**) After randomization, mice were treated with vehicle (*n*=4) cetuximab (*n*=4), pimasertib (*n*=4) or the combination (*n*=4) for 6 weeks after which drug treatment was stopped and restarted (red arrows and black line). Treatment with vehicle served as control. Error bars represent s.e.m. (**b**) Individual mice from cetuximab or combo treated cohorts are represented as percentage of single tumours shrinkage on treatment at three time points after treatment start.

To assess more directly the clinical relevance of our findings we employed a patient-derived xenograft (PDX or xenopatient) sensitive to EGFR blockade. This was derived from an individual carrying a quadruple wild-type (*KRAS*, *NRAS*, *BRAF* and *PIK3CA*) colorectal tumour, and thus recapitulates the molecular profile of patients sensitive to anti-EGFR antibodies. To measure the TTP *in vivo*, the xenograft was first expanded in four cohorts that were treated for 6 weeks with vehicle, cetuximab, the MEK inhibitor pimasertib or the combination. As observed *in vitro*, MEK blockade had a marginal effect on tumour growth while cetuximab and combinatorial treatments significantly reduced tumour size in all animals. By week 7 tumours treated with cetuximab had shrunk by 70% but were still palpable; on the contrary those who received EGFR-MEK combo blockade were undetectable (Fig. 3a,b and Supplementary Data 1). To assess whether treatments had been curative, at week 9 both regimens were suspended while tumour sizes continued to be monitored. Tumours that had previously received only cetuximab immediately begun to regrow while those that had been treated with EGFR and MEK inhibitors remained undetectable or showed marginal growth by week 15 (Fig. 3a,b and Supplementary Data 1). At this point both drug schedules were restarted. Tumours that previously received cetuximab alone momentarily halted their expansion on drug re-challenge and then swiftly resumed growth. On the contrary, those tumours treated with the combo regimen remained undetectable until week 28.

To further validate the *in vivo* results, we repeated the experiment with the PDX avoiding treatment interruption. This second replicate, confirmed the results obtained in the first experiment, emphasizing the finding that combinatorial treatment allow complete xeno-tumour remission (Supplementary Fig. 4a and Supplementary Data 2).

The same experimental strategy was then applied to an independent PDX that was also derived from a quadruple wild-type (*KRAS*, *NRAS*, *BRAF* and *PIK3CA*) colorectal tumour and was sensitive to EGFR blockade. Analogously to what we observed in the first xenopatient the effect of monotherapy with cetuximab was transient and secondary resistance developed at week 16 of treatment (Supplementary Fig. 4b–c). On the contrary secondary resistance was not detected in the combo treated arm until week 23 when the experiment was terminated (Supplementary Fig. 4b–c and Supplementary Data 3).

### Mathematical modelling of drug resistance in PDX

It has been postulated that the limited efficacy of therapies based on single agents is related to the presence of drug-resistant cells at the start of treatment^12^,^17^,^19^,^20^,^21^,^22^,^23^,^24^. We reasoned that the xenopatients' data could offer a unique opportunity to study the size of this resistant subpopulation and quantify the dynamics of CRC treated with mono or combo therapies. To this end, we used mathematical modelling to analyse the experimental data. We started by fitting the average volume of tumours from the replicate experiment treated with cetuximab (Supplementary Data 2) to a cancer dynamics model, which assumes that tumours contain a mix of sensitive and resistant cells at the time of treatment initiation (Supplementary Methods):


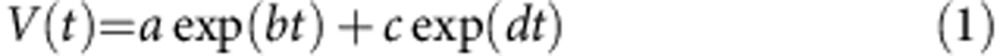


**Figure 4:**
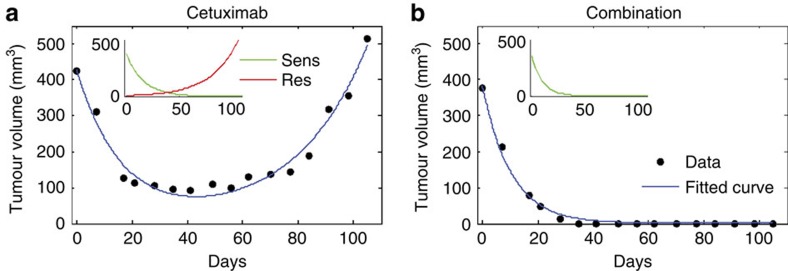
Mathematical modelling of cetuximab and combinatorial treatment *in vivo*. (**a**) Fit of model (equation 1 described in the previous page) to average tumour volume of PDX1 cetuximab-treated replicate experiment. Fitted curve (blue) is the sum of sensitive (green) and resistant (red) populations, whose predicted behaviours are shown in inset. (**b**) Fit of model (equation 2 described in the previous page) to average tumour volume of PDX1 combo treated replicate experiment.

Here *a* is the volume of sensitive cells in the tumour at time 0 (start of treatment) and *b*<0 is their (fixed) net growth rate during treatment. *c* is the volume of resistant cells at day 0 and *d*>0 is their (fixed) net growth rate during treatment. The model assumes deterministic exponential growth and independence of sensitive and resistant clones, and further assumes that drugs have constant effect on cells throughout the duration of treatment. This simple model fits the data well (*R*^2^=0.97, Fig. 4a) without the need to invoke additional assumptions. As described in the Supplementary Methods the assumptions of exponential growth and independence of clones become violated in large tumours (with volumes larger than ∼1,000 mm^3^) when growth slows down and turns from exponential to logistic (Supplementary Fig. 5a,b).

The best fit to cetuximab-treated xenopatient data was obtained with a model in which the volume of resistant cells at the start of treatment is *c*=8.8 (95% CI, 2.8–14.8) mm^3^ and the volume of sensitive cells is *a*=417.2(95% CI, 365–469.5) mm^3^. In other words, ∼2% of all cells present in the tumour at the start of treatment are resistant to cetuximab and they grow during treatment at rate *d*=0.038(95% CI, 0.031–0.045) per day. Sensitive cells decline at rate *b*=0.062(95% CI, −0.077 to −0.046) per day during treatment (Supplementary Table 2).

The original xenopatient experiment included cessation of treatment, and, by fitting these data to a more complicated mathematical model that included four phases of treatment and its absence (Supplementary Methods and Supplementary Fig. 5c), we found that the growth rate of resistant cells in the absence of treatment was *e*=0.028 (95% CI, 0.017–0.038) per day, while the growth rate of sensitive cells in the absence of treatment was *f*=0.078 (95% CI, 0.041–0.12) per day. This means that resistance is costly in the absence of treatment as it decreases the net growth rate by ∼0.05 per day. The relatively high percentage of resistant cells at the start of treatment in spite of the cost of resistance can be explained by a relatively high rate of production of resistant cells from the sensitive population before treatment. It can be shown that disadvantageous cells that are produced with rate u, and which decrease the net growth rate by a cost z, are present in the population at an average fraction of u/z (mutation-selection balance). It follows that the rate of production of resistant cells is on the order of 0.001 per day. This rate is much higher than if resistance was mediated by ∼100 point mutations, when it would be ∼10^–7^, indicating that the mechanism of resistance is not mediated by point mutations only, but likely via other genetic or non-genetic mechanisms.

In the case of EGFR-MEK combinatorial treatment, we see no evidence of resistant cells in tumour volume data, and thus we fit a model that contains only sensitive cells. The model assumes that sensitive cells decline with rate *b*<0 during treatment (Supplementary Methods and Supplementary Table 3):


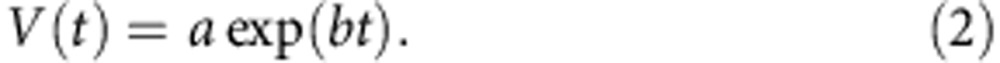


Volume of sensitive cells at the start of therapy (day 0) is *a*. Again this model fits the xenopatient data well (*R*^2^=0.99; Fig. 4b). We find that sensitive cells decline with rate *b*=−0.096 (−0.10, −0.088) during combinatorial treatment. These results suggest that there were still approximately a million sensitive cells in the tumour when the treatment in the original experiment was stopped and these sensitive cells grew back in the absence of treatment (Fig. 3a, Supplementary Fig. 5d). In addition, fitting the tumour volume data from the original experiment with combinatorial treatment, we find that cells sensitive to combination treatment grow slower after being exposed to the treatment compared with untreated sensitive cells. The growth rate of combination-treated cells in the absence of therapy is 0.05 (95% CI 0.037–0.062) per day versus 0.085 (95% CI 0.074–0.096) per day for untreated cells. For consistency, we also applied our model to the second PDX (Supplementary Methods, Supplementary Fig. 5e,f and Supplementary Table 4).

**Figure 5:**
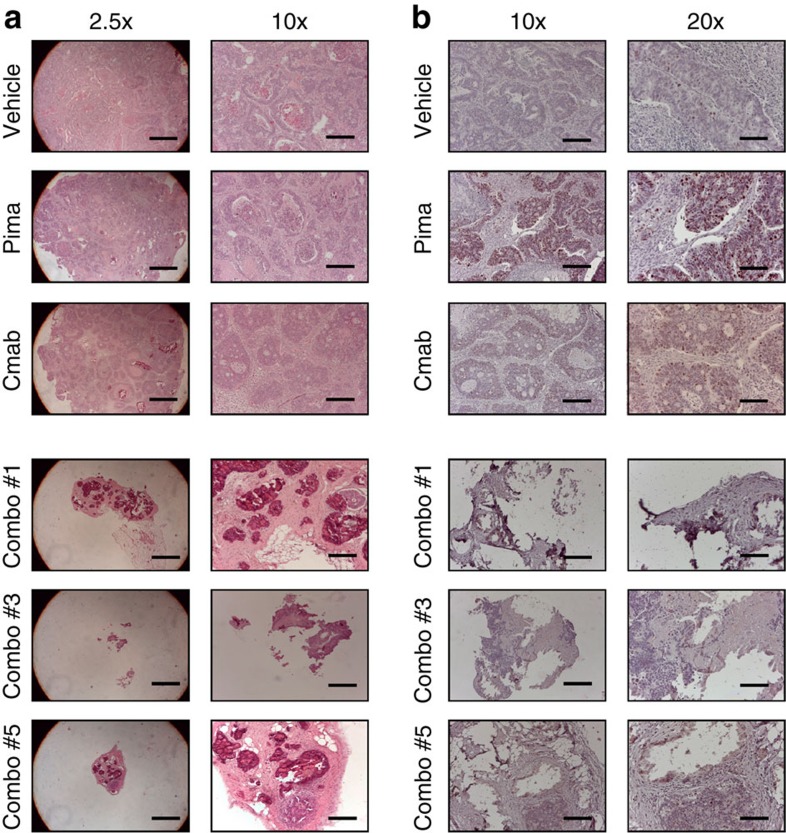
Immunohystochemical analyses of PDX derived samples. Two levels of magnification are presented to better appreciate sample size. (**a**) Ki67 staining of one representative example of Vehicle, Pima and Cmab treated mice and the entire combo treated mice from which we were able to obtain tissue at the end of the experiment. Percentage of positivity: Vehicle 6%, Pima 24%, Cmab 8% and Combo 0%. (**b**) Hematoxylin/Eosin staining of one representative example of Vehicle, Pima and Cmab treated mice and the entire combo treated mice from which we were able to obtain tissue at the end of the experiment. Only in mouse #5 a small amount of cancer cells is detectable, the rest are composed only by necrotic tissue. Scale bars, 50 μm (**a**, right panels), 100 μm (**a**, lefts panel; and **b**, right panels) and 500 μm (**b**, left panels).

Given these results, there is no evidence in xenograft experiments of the existence of dual-resistant cells. To better understand this, we performed immunohystochemical analyses on tumour sections derived from the first PDX model. Using this approach we were able to determine that the tumour ‘scar', which is present at the end of the experiment in the combo treated mice corresponds essentially to necrotic tissue. Even when a few tumour cells appear to be still present, they are not proliferating as shown by Ki67 staining (Fig. 5). Taking into account the last non-zero volume measurement (13.5 mm^3^ or ∼13.5 million cells) of the tumour from the mouse in which a small number of non-proliferative tumour cells were found, the time from this measurement to the end of the experiment (84 days) and the estimated death rate of sensitive cells during combo treatment (−0.096 per day), it is expected that ∼4,000 sensitive cells were still present at the end of the experiment that have not yet been killed by the treatment. Thus, we postulate that the small amount of non-proliferative cells found in one of the combo treated mice are likely sensitive cells that are not yet killed by the treatment (Fig. 5). Total tumour volume in the four mice treated with the combination is ∼1,600 mm^3^ or ∼1.6 billion tumour cells. Since none of the four mice treated with the combination harboured any surviving cells resistant to the combo, we can conclude that the frequency of pre-existing successful combo-resistant cells is <1 in a billion. It is possible that each tumour contained a small number of combo-resistant or persistor cells, which were not sufficient to form a surviving resistant population, or that died due to stochastic drift.

### Mathematical modelling of drug resistance in cell models

**Figure 6:**
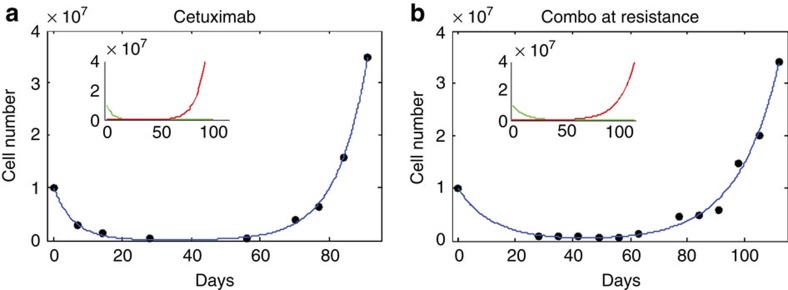
Mathematical modelling of cetuximab and combinatorial treatment *in vitro*. (**a**), Fit of model (equation 1) to cell number data of DiFi cell line treated with cetuximab. (**b**) Fit of model (equation 2) to the cell number data of DiFi cetuximab-resistant population re-challenged with the combo.

Since it was impossible to assess the fitness of cells resistant to combinatorial treatment from the xenopatient experiments, we turned to *in vitro* experiments in which double EGFR-MEK-resistant mutants appeared in a population that was already resistant to cetuximab. We first fit the cell number data from a DiFi cell line treated with cetuximab to the same model used for fitting xenopatient tumours treated with cetuximab (equation 1). The only difference is that now *a* and *c* are cell numbers and not volumes. The fit of the model to the data was excellent (*R*^2^=0.999, Fig. 6a). Out of 10 million cells that were present at the start of treatment, only *c*=1,077 (95% CI, 240–1,915) were resistant to cetuximab. These resistant cells expanded during treatment at rate *d*=0.114 (95% CI, 0.105–0.123) per day. Sensitive cells were declining during treatment at rate *b*=−0.165 (95% CI, −0.217 to 0.113) per day. Ten million cells that developed resistance to cetuximab were plated and treated with combination of cetuximab and pimasertib. The same model (1) was again an excellent fit to the cell number data (*R*^2^=0.99; Fig. 6b). Out of 10 million initial cells, *c*=1.5·10^4^ (95% CI, 550–3 × 10^4^) are predicted to have been resistant to the combination at the start of treatment. Their growth rate during treatment was *d*=0.069 (95% CI, 0.060–0.078) per day, smaller compared with the growth rate of cells resistant to cetuximab only. In summary, ∼1 in 10,000 DiFi cells are resistant to cetuximab; out of cetuximab-resistant cells, ∼1 in 1,000 are resistant to the combo but they emerged only on the acquisition of cetuximab resistance.

Our modelling results show that the frequency of pre-existing combo-resistant cells may vary in different cell lines and patient tumours. Thus, a typical metastatic lesion that contains about a billion cells may not have a single combo-resistant cell or it may contain a small number of combo-resistant cells. The small number of combo-resistant cells may not be sufficient to form a successful resistant population (due to, for example, stochastic drift)^12^, and in fact we never see resistance to combination therapy in our experiments when the EGFR-combo is applied on naive cells (as front line therapy). In contrast, when single therapy such as cetuximab is the first line of treatment, resistance inevitably develops and this cetuximab-resistant subpopulation is much more likely to harbour a combo-resistant mutant. In sum, both theoretical predictions^12^ and our experiments strongly favour combination of EGFR and MEK combo blockade as first line treatment of CRC patients.

### Adaptive escape from MEK inhibition in CRC cells

**Figure 7:**
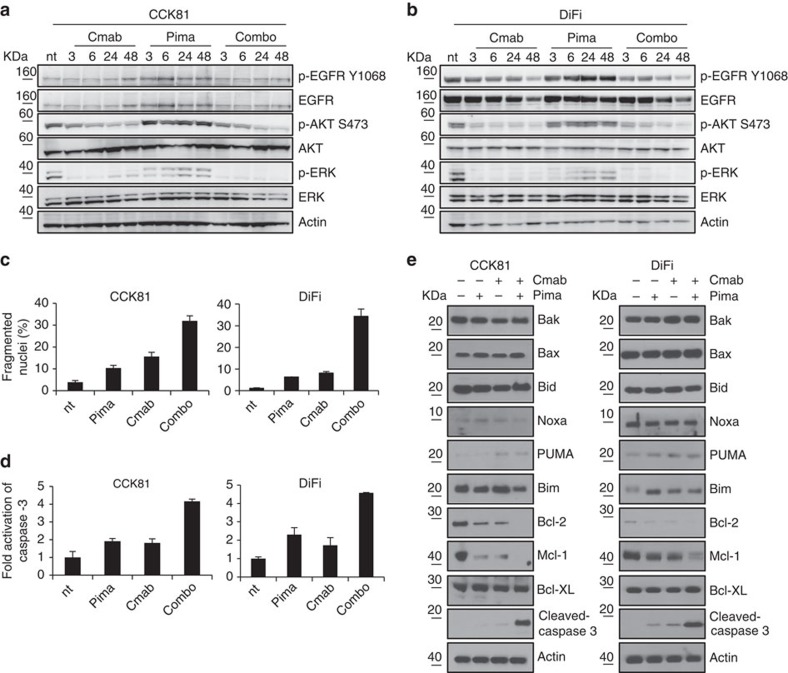
Biochemical analysis and apoptosis evaluation of CRC cells treated with EGFR and/or MEK inhibitors. CCK81 (**a**) and DiFi (**b**) were treated with cetuximab (Cmab, 340 nM), pimasertib (Pima, 250 nM), or with the combo of the two drugs at the indicated time points, whole-cell extracts were subjected to western blot analysis and compared with untreated cells with phospho-EGFR (Tyr 1068), total EGFR, total AKT and phospho-AKT (Ser 473), total ERK1/2 and phospho-ERK1/2 antibodies. Actin was included as a loading control. (**c**,**d**) Concomitant blockade of EGFR and MEK triggers apoptosis. CCK81 and DiFi cells were treated at different time points with cetuximab, pimasertib or both. Nuclei fragmentation (**c**) and caspase-3 activation (**d**) were measured. Rates of apoptosis due to combination are approximatively twofold higher compared with those due to cetuximab, in line with our model predictions. (**e**) Apoptotic pathway activation. The indicated CRC cell lines were treated with cetuximab (Cmab, 340 nM), pimasertib (Pima, 250 nM), or with the two drugs for 48 h. Whole-cell extracts were subjected to Western blot analysis and compared with untreated cells using BAK, Bax, Bid, NOXA, PUMA, Bim, Bcl-2, Mcl-1, Bcl-XL and active caspase-3 antibodies. Actin was included as a loading control.

Experiments in cells and tumourgrafts and mathematical models indicate that combinatorial blockade of EGFR and MEK impairs the emergence of resistant clones *in vitro* and *in vivo*. At first this tactic may seem counter-intuitive. Why should it be necessary to intercept two nodes in the same pathway? To shed light on the remarkable efficacy of EGFR-MEK concomitant inhibition we performed biochemical analyses. The experiments of Fig. 2 indicate that CRC cells sensitive to EGFR blockade are virtually unaffected by MEK inhibition and quickly resume exponential growth in the presence of MEK suppression. Biochemical profiling of EGFR downstream pathways in DiFi and CCK81 cells revealed that MEK inhibition induces EGFR and AKT activation (p-Y1068 and p-S473) and is unable to switch off ERK phosphorylation, thus suggesting an adaptive escape from MEK blockade (Fig. 7a,b and Supplementary Fig 7a,b).

### Combinatorial therapy triggers apoptosis in CRC models

Results from fitting xenopatients data suggest that the supremacy of combination versus single drug treatments is twofold: the combination is not opposed by pre-existing resistance and is more effective in killing cancer cells as compared with cetuximab (net death rate 0.96 (95% CI, 0.088–0.10) versus 0.62 (95% CI, 0.046–0.077) per day). To test whether the combination is more effective in triggering apoptosis than monotherapy, we challenged DiFi and CCK81 cells with mono or combo treatments and monitored indicators of programmed cell death. In both cell lines the proportion of fragmented nuclei (Fig. 7c and Supplementary Fig. 7c) and of cleaved caspases-3 (Fig. 7d and Supplementary Fig. 7d) was higher in combo treated cells compared with the other conditions. We further found that combinatorial treatment is capable of inducing apoptosis by triggering a significant decrease of Bcl-2 and Mcl-1 (ref. 25) expression (Fig. 7e and Supplementary Fig. 7e). Additional analyses showed that downregulation is mediated by protein degradation through enhanced ubiquitination of Mcl-1 and by transcriptional down modulation of Bcl-2 (Supplementary Fig. 7 and Supplementary Fig. 8). Altogether, these data show that EGFR-MEK combo treatment induces apoptosis at a rate that is approximately twofold higher compared with cetuximab monotherapy (Fig. 6a,b), consistent with predictions from mathematical modelling of the xenopatient data.

## Discussion

Analysis of candidate genes in CRC patients' samples previously indicated that acquired resistance to EGFR blockade is associated with the emergence of clones carrying oncogenic mutations in the EGFR-RAS signalling axis^16^,^17^,^26^,^27^,^28^,^29^. Biochemical studies showed that resistance to EGFR blockade is almost invariably accompanied by activation (constitutive phosphorylation) of MEK and ERK^29^. Through a genetic screen involving 27 pathway-activating proteins we now provide unbiased functional and biochemical evidence that constitutive activation of RAS-MAPK signalling (but not of other pathways) is sufficient to confer resistance to EGFR blockade. The multitude of evidences suggesting that escape routes from EGFR blockade in CRC biochemically convergence on MAPK activation provides several opportunities, which we exploited in this work.

Using an assay that recapitulates TTP *in vitro* and *in vivo*, we find that vertical suppression of the EGFR signalling pathway halts or prevents the onset of acquired resistance in CRC cell and xenopatients. Perhaps not unexpectedly when the same therapeutic regimen is applied once resistance to monotherapy has already occurred, the efficacy of the EGFR-MEK combo is only transient. These results provide support for ‘*ab initio*' combinatorial therapies, which conceivably, could restrain the development of resistant tumours displaying high levels of molecular heterogeneity.

Mathematical modelling was used to quantify the dynamics of sensitive and resistant clones, showing that clones resistant to combination are unlikely to exist before treatment. Our modelling approach provides a framework for comparing and contrasting the long-term effects of cancer therapies *in vitro* and *in vivo*. The mathematical analysis was focused on pre-existing resistance, even though other mechanisms of resistance might be involved, such as resistance arising *de novo* during treatment. Several studies tried to estimate the significance of pre-existing versus *de novo* resistance to therapy in cancer and in viral infections, and often found that resistance mainly arises before treatment^16^,^17^,^19^,^21^.

Of note, emergence of double resistant clones was observed only when single agent resistant populations were allowed to develop and then treated with the combo regimen. Acquisition of resistance to combinatorial therapy could thus potentially be enabled by genetic or non-genetic mechanisms and enhanced during cetuximab treatment. It also may be influenced by stochastic drift^12^ or clonal interference^30^ as well as by the presence of so called ‘persistor cells^31^. Further investigations are needed to better understand the mechanistic basis of resistance to combo EGFR-MEK blockade.

Biochemical analyses revealed that vertical suppression of the EGFR-MEK signalling pathway triggers apoptosis at a higher rate (approximately twofold), compared with cetuximab alone. On the basis of these results we propose that CRC cells without RAS pathway mutations do not survive in the absence of continuous signals coordinated by EGFR and MEK. We postulate that the inability to develop resistance *in vitro* and *in vivo* stems from suppression of a central (essential) pathway in CRC cells lacking KRAS mutations. In this regard, the vertical blockade described here may be the biochemical equivalent of synthetic lethality. We further imply that EGFR-MEK concomitant inhibition likely suppresses the emergence of resistant clones by subduing the population of molecularly heterogeneous cancer cells^32^, which fuels the relative rapid recurrences observed after treatments based on EGFR antibodies.

In summary our results highlight that integration of clinical data with functional analyses of preclinical models can lead to rational therapies aimed at delaying or halting the emergence of drug resistance in cancer cells. Our data indicate that EGFR/MEK combo blockade is better than upfront treatment with anti-EGFR antibodies or to delivering a MEK inhibitor when resistance to cetuximab or panitumumab has already developed. As several EGFR and MEK inhibitors are approved or in advanced clinical development (alone or in combination) our results provide grounds for clinical trials aimed at preventing rather than intercepting resistance.

## Methods

### Cell cultures

DiFi cells were cultured in F12 medium (Invitrogen) supplemented with 10% fetal bovine serum (FBS); CCK81 cells were cultured in MEM medium (Invitrogen) supplemented with 10% FBS. LIM1215 cells were cultured in RPMI-1640 medium (Invitrogen) supplemented with 5% FBS and insulin (1 μg ml^−1^). CCK81 cell line was obtained from HSRRB, Japan. The DiFi cell line was a kind gift from Dr J. Baselga in November 2004 (Oncology Department of Vall d'Hebron University Hospital, Barcelona, Spain) and Dr V. Cerundolo in March 2010 (Weatherall Institute of Molecular Medicine, University of Oxford, UK), respectively. C99 cells were cultured in Iscove's medium (Invitrogen) supplemented with 10% FBS; NCIH508 cells were cultured in RPMI-1640 medium (Invitrogen) supplemented with 10% FBS, and HCA-46 cells were cultured in Dulbecco's modified Eagle's medium (Invitrogen) supplemented with 10% FBS. The NCIH508 cell line was purchased from American Type Culture Collection (LGC Standards S.r.l). CCK81 cell line was obtained from HSRRB, Japan. C99 and HCA-46 cell lines were obtained from ECACC (distributed by Sigma-Aldrich Srl). The LIM1215 parental cell line have been described previously^33^ and was obtained from Professor Robert Whitehead, Vanderbilt University, Nashville, with permission from the Ludwig Institute for Cancer Research, Zurich, Switzerland. The identity of each cell line was checked by Cell ID System and by Gene Print 10 System (Promega), through short tandem repeats (STR) at 10 different loci (D5S818, D13S317, D7S820, D16S539, D21S11, vWA, TH01, TPOX, CSF1PO and amelogenin). Amplicons from multiplex PCRs were separated by capillary electrophoresis (3730 DNA Analyzer, Applied Biosystems) and analysed using GeneMapperID software from Life Technologies. Resulting cell line STR profiles were cross-compared and matched with the available STR from ATC, ECCAC, and CellBank Australia repositories online databases. All cell lines were tested and resulted negative for mycoplasma contamination with Venor GeM Classic kit (Minerva biolabs).

### TTP assay

For the TTP long-term assay, 20 million CCK81 and C99, and 10 million DiFi, NCIH508 and HCA-46 cell lines were plated in their respective growth media with half serum (5%) and treated with cetuximab (340 nM), pimasertib, (250 nM) or the combination of the two. Cells were counted every 4 days (CCK81 day 10 to day 70) or every week (all the other time points and cell lines). Count as 0 represent time points in which cells were too few and only medium and drug refreshments were done.

### Immunoblot analysis

Before biochemical analysis, all cells were grown in their specific media supplemented with 5% FBS. Total cellular proteins were extracted by solubilizing the cells in cold EB buffer (50 mM Hepes pH 7.4, 150 mM NaCl, 1% Triton X-100, 10% glycerol, 5 mM EDTA, 2 mM EGTA; all reagents were from Sigma-Aldrich, except for Triton X-100 from Fluka) in the presence of 1 mM sodium orthovanadate, 100 mM sodium fluoride and a mixture of protease inhibitors (pepstatin, leupeptin, aprotinin, STI and phenylmethylsulfonyl fluoride). Extracts were clarified by centrifugation, and protein concentration was determined using BCA protein assay reagent kit (Thermo). Western blot detection was performed with enhanced chemiluminescence system (GE Healthcare) and peroxidase conjugated secondary antibodies (Amersham). The following primary antibodies were used for western blotting (all from Cell Signaling Technology, except where indicated): anti-phospho-p44/42 ERK (thr202/tyr204); anti-p44/42 ERK; anti-phospho-MEK1/2 (Ser217/221), anti-MEK1/2; anti-phospho-AKT (Ser473), anti-AKT, anti-EGFR (clone13G8, Enzo Life Sciences); anti-phospho-EGFR (tyr1068); anti-actin and anti-vinculin (Sigma-Aldrich), anti-PUMA, anti-Bid, anti-cleaved-caspase-3 (Cell Signaling Technology), anti-Mcl-1 (Santa Cruz Biotechnology), anti-Bak, anti-Bcl-2, anti-Bim, anti-Noxa (EMD Millipore), and anti-Bcl-XL (BD Biosciences). All the antibodies were diluted 1:1,000 except for total EGFR which was diluted 1:100. Mcl-1 ubiquitination was analysed by probing immunoprecipitated Mcl-1 in cells transfected with HA-tagged ubiquitin and treated with drugs at 6 h after transfection as described^34^.

### Apoptosis analysis

After drug treatment, adherent and floating cells were collected and resuspended with PBS solution containing 3.7% formaldehyde, 0.5% Nonidet P-40, and 10 μg ml^−1^ Hoechst 33258 (Invitrogen). Apoptosis was assessed through microscopic visualization and counting of cells with condensed chromatin and micronucleations. For each measurement, at least three independent experiments and a minimum of 300 cells were analysed. Caspase activity was measured using the SensoLyte Homogeneous AMC Caspase-3/7 Assay Kit (Anaspec) according to the manufacturer's instructions. Briefly, 5.0 × 10^4^ DiFi or CCK81 cells were seeded in each well of a 96-well plate. After treatment, cells were incubated with the caspase substrate Ac-DEVD-AMC at room temperature for 40 min. Fluorescence was measured using a Wallac Victor 1420 Multilabel Counter (PerkinElmer), and the data were presented as relative fluorescence units.

### Real-Time PCR

Total RNA was isolated from 3.0 × 10^5^ cells using Mini RNA Isolation II Kit (Zymo Research) according to the manufacturer's protocol. One microgram of total RNA was used to generate cDNA using SuperScript II reverse transcriptase (Invitrogen). Real-time RT–PCR was performed on a CFX96 Real-Time PCR system (Bio-Rad, Hercules, CA) with SYBR Green (Invitrogen). Bcl-2 and Mcl-1 were analysed by RT–PCR using primer pairs 5′- ATGTGTGTGGAGAGCGTCAACC -3′/5′- TGAGCAGAGTCTTCAGAGACAG -3′ and 5′- ATGCTTCGGAAACTGGACAT -3′/5′- TGGAAGAACTCCACAAACCCA -3′, respectively, as previously described^34^.

### GI50 assays with pathway-activating library

For details on the cloning, sequencing and functional validation of the library of pathway-activating lentiviral cDNAs, refers to Martz *et al*.^18^, Science Signaling 2014. cDNA-expressing lentiviruses were produced as previously described^35^ and used to infect cells at a 1:10–1:20 dilution in 6-well plates in the presence of 7.5 μg ml^−1^ polybrene. Following virus addition, plates were centrifuged at 1,200*g* for 1 h at 37 °C. Twenty-four hours after infection, puromycin (2 μg ml^−1^) was added for selection and cells were incubated for 48–72 h. Cells were then trypsinized, counted, and seeded into 96-well plates at 1,000 cells/well. Twenty-four later, media or tenfold serial dilutions of panitumumab (in media) were added to cells (1:1,000) to yield final concentrations ranging from 680 nM to 0.000068, nM. The Cell Titer Glo luminescent viability assay (Promega) was used to measure cell viability seven days after drug addition. Viability was calculated as the percentage of control (untreated cells) after background subtraction with a minimum of three replicates for each cell line/cDNA/drug concentration. GI50 values were determined as the drug dose corresponding to half-maximal growth inhibition^35^; as such, the upper bound GI50 value in this assay is 680 nM. GI50 values for unmodified parental cells were determined using the protocol above by seeding cells directly into 96-well plates without the initial infection step.

### Patient-derived xenograft

The first patient was diagnosed with stage IV (metastatic) CRC in 2006. Surgical material derived from a second metastatic relapse in the liver in 2009 was collected, cut into pieces and implanted in NOD-SCID mice. Early passage specimens were used for this study. After engraftment, the tumour was passaged and expanded for two generations until production of four cohorts. These were randomized according to average tumour size of 400 mm^3^ and treated with vehicle alone (four mice), cetuximab monotherapy (five mice), pimasertib monotherapy (four mice), or their combination (five mice). The investigator was not blinded. Two mice, one in the cetuximab arm and the other in the combo arm died during treatment for reasons unrelated with pharmacological treatments and were therefore excluded from subsequent analyses. Treatments started at the second week and lasted 6 weeks. At this point, treatments were stopped and restarted after 6 weeks. Animals receiving vehicle, or pimasertib alone had to be euthanized after 5 and 6 weeks of treatment for ethical reasons. A second replicate of this PDX model was performed without vehicle and pimasertib treated arms and without stopping the treatment with cetuximab (six mice) or cetuximab plus pimasertib combination (eight mice). The second patient was diagnosed with metastatic relapse from rectal cancer in 2008. Surgical material derived from resection of a liver metastasis in 2009 was collected under adequate informed consent, cut into pieces and implanted in NOD-SCID mice. Early passage specimens were used also in this case. After engraftment, the tumour was passaged and expanded for two generations until production of four cohorts. These were randomized according to average tumour size of 400 mm^3^ and treated with vehicle alone (four mice), cetuximab monotherapy (six mice), pimasertib monotherapy (five mice), or their combination (six mice). Caliper measurements were taken once a week. Cetuximab was given by intraperitoneal injection at 0.5 mg kg^−1^ twice a week, and pimasertib was administered by gavage at 50 mg kg^−1^ per day. Pimasertib was resuspended in distilled sterile water containing 0.5% carboxymethylcellulose (Sigma Aldrich) and 0.25% Tween 80 (Sigma Aldrich). All animal procedures were approved by the Ethical Commission of the Institute for Cancer Research and Treatment and by the Italian Ministry of Health. Informed consent for research use of patient tumour material was obtained from patients at the Candiolo Cancer Institute prior to tissue banking and study approval was obtained from the ethics committee of the enroling institution.

### Immunohystochemical analyses

Xenografts were embedded in paraffin and subjected to hematoxylin-and-eosin staining or to immunoperoxidase staining with the mouse monoclonal anti-Ki-67 antibody (clone MIB-1, Dako). After incubation with secondary antibodies, immunoreactivities were revealed by incubation in DAB chromogen (Dako). Images were captured with the Leica LAS EZ software using a Leica DM LB microscope.

## Additional information

**How to cite this article**: Misale, S. *et al*. Vertical suppression of the EGFR pathway prevents onset of resistance in colorectal cancers. *Nat. Commun.* 6:8305 doi: 10.1038/ncomms9305 (2015).

## Supplementary Material

Supplementary Information Supplementary Figures 1-8, Supplementary Tables 1-4 and Supplementary Methods

Supplementary Data 1 Tumours' measurements of first PDX model.

Supplementary Data 2 Tumours' measurements of second replicate of the first PDX model.

Supplementary Data 3 Tumours' measurements of the second PDX model.

## References

[b1] Vogelstein B. & Kinzler K. W. Cancer genes and the pathways they control. Nat. Med. 10, 789–799 (2004). 15286780 10.1038/nm1087

[b2] Pines G., Köstler W. J. & Yarden Y. Oncogenic mutant forms of EGFR: lessons in signal transduction and targets for cancer therapy. FEBS Lett. 584, 2699–2706 (2010). 20388509 10.1016/j.febslet.2010.04.019 PMC2892754

[b3] Weinstein I. B. Cancer. Addiction to oncogenes--the achilles heal of cancer. Science 297, 63–64 (2002). 12098689 10.1126/science.1073096

[b4] Misale S., Di Nicolantonio F., Sartore-Bianchi A., Siena S. & Bardelli A. Resistance to anti-EGFR therapy in colorectal cancer: from heterogeneity to convergent evolution. Cancer Discov. 4, 1269–1280 (2014). 25293556 10.1158/2159-8290.CD-14-0462

[b5] Chong C. R. & Jänne P. A. The quest to overcome resistance to EGFR-targeted therapies in cancer. Nat. Med. 19, 1389–1400 (2013). 24202392 10.1038/nm.3388 PMC4049336

[b6] Poulikakos P. I. & Rosen N. Mutant BRAF melanomas--dependence and resistance. Cancer Cell 19, 11–15 (2011). 21251612 10.1016/j.ccr.2011.01.008

[b7] Engelman J. A. . MET amplification leads to gefitinib resistance in lung cancer by activating ERBB3 signaling. Science 316, 1039–1043 (2007). 17463250 10.1126/science.1141478

[b8] Pao W. . Acquired resistance of lung adenocarcinomas to gefitinib or erlotinib is associated with a second mutation in the EGFR kinase domain. PLoS Med. 2, e73 (2005). 15737014 10.1371/journal.pmed.0020073 PMC549606

[b9] Girotti M. R. . Inhibiting EGF receptor or SRC family kinase signaling overcomes BRAF inhibitor resistance in melanoma. Cancer Discov. 3, 158–167 (2013). 23242808 10.1158/2159-8290.CD-12-0386 PMC5321574

[b10] Wagle N. . Dissecting therapeutic resistance to RAF inhibition in melanoma by tumor genomic profiling. J. Clin. Oncol. 29, 3085–3096 (2011). 21383288 10.1200/JCO.2010.33.2312 PMC3157968

[b11] Burrell R. A. & Swanton C. Tumour heterogeneity and the evolution of polyclonal drug resistance. Mol. Oncol. 8, 1095–1111 (2014). 25087573 10.1016/j.molonc.2014.06.005 PMC5528620

[b12] Bozic I. . Evolutionary dynamics of cancer in response to targeted combination therapy. Elife 2, e00747 (2013). 23805382 10.7554/eLife.00747 PMC3691570

[b13] Glickman M. S. & Sawyers C. L. Converting cancer therapies into cures: lessons from infectious diseases. Cell 148, 1089–1098 (2012). 22424221 10.1016/j.cell.2012.02.015 PMC3465702

[b14] Flaherty K. T. . Combined BRAF and MEK inhibition in melanoma with BRAF V600 mutations. N. Engl. J. Med. 367, 1694–1703 (2012). 23020132 10.1056/NEJMoa1210093 PMC3549295

[b15] Douillard J. Y. . Final results from PRIME: randomized phase 3 study of panitumumab with FOLFOX4 for first-line treatment of metastatic colorectal cancer. Ann. Oncol. 25, 1346–1355 (2014). 24718886 10.1093/annonc/mdu141

[b16] Misale S. . Emergence of KRAS mutations and acquired resistance to anti-EGFR therapy in colorectal cancer. Nature 486, 532–536 (2012). 22722830 10.1038/nature11156 PMC3927413

[b17] Diaz L. A. . The molecular evolution of acquired resistance to targeted EGFR blockade in colorectal cancers. Nature 486, 537–540 (2012). 22722843 10.1038/nature11219 PMC3436069

[b18] Martz C. A. . Systematic identification of signaling pathways with potential to confer anticancer drug resistance. Sci. Signal. 7, ra121 (2014). 25538079 10.1126/scisignal.aaa1877 PMC4353587

[b19] Komarova N. L. & Wodarz D. Drug resistance in cancer: principles of emergence and prevention. Proc. Natl Acad. Sci. USA 102, 9714–9719 (2005). 15980154 10.1073/pnas.0501870102 PMC1172248

[b20] Bozic I., Allen B. & Nowak M. A. Dynamics of targeted cancer therapy. Trends Mol. Med. 18, 311–316 (2012). 22595628 10.1016/j.molmed.2012.04.006 PMC3372676

[b21] Bozic I. & Nowak M. A. Timing and heterogeneity of mutations associated with drug resistance in metastatic cancers. Proc. Natl Acad. Sci. USA 111, 15964–15968 (2014). 25349424 10.1073/pnas.1412075111 PMC4234551

[b22] Leder K. . Fitness conferred by BCR-ABL kinase domain mutations determines the risk of pre-existing resistance in chronic myeloid leukemia. PLoS One 6, e27682 (2011). 22140458 10.1371/journal.pone.0027682 PMC3225363

[b23] Michor F. . Dynamics of chronic myeloid leukaemia. Nature 435, 1267–1270 (2005). 15988530 10.1038/nature03669

[b24] Iwasa Y., Nowak M. A. & Michor F. Evolution of resistance during clonal expansion. Genetics 172, 2557–2566 (2006). 16636113 10.1534/genetics.105.049791 PMC1456382

[b25] Czabotar P. E., Lessene G., Strasser A. & Adams J. M. Control of apoptosis by the BCL-2 protein family: implications for physiology and therapy. Nat. Rev. Mol. Cell Biol. 15, 49–63 (2014). 24355989 10.1038/nrm3722

[b26] Bettegowda C. . Detection of circulating tumor DNA in early- and late-stage human malignancies. Sci. Transl. Med. 6, 224ra224 (2014). 10.1126/scitranslmed.3007094 PMC4017867 24553385

[b27] Bardelli A. . Amplification of the MET receptor drives resistance to anti-EGFR therapies in colorectal cancer. Cancer Discov. 3, 658–673 (2013). 23729478 10.1158/2159-8290.CD-12-0558 PMC4078408

[b28] Bertotti A. . A molecularly annotated platform of patient-derived xenografts ("xenopatients") identifies HER2 as an effective therapeutic target in cetuximab-resistant colorectal cancer. Cancer Discov. 1, 508–523 (2011). 22586653 10.1158/2159-8290.CD-11-0109

[b29] Misale S. . Blockade of EGFR and MEK intercepts heterogeneous mechanisms of acquired resistance to anti-EGFR therapies in colorectal cancer. Sci. Transl. Med. 6, 224ra226 (2014). 10.1126/scitranslmed.3007947 24553387

[b30] Polyak K. & Marusyk A. Cancer: clonal cooperation. Nature 508, 52–53 (2014). 24695309 10.1038/508052a

[b31] Sharma S. V. . A chromatin-mediated reversible drug-tolerant state in cancer cell subpopulations. Cell 141, 69–80 (2010). 20371346 10.1016/j.cell.2010.02.027 PMC2851638

[b32] Burrell R. A., McGranahan N., Bartek J. & Swanton C. The causes and consequences of genetic heterogeneity in cancer evolution. Nature 501, 338–345 (2013). 24048066 10.1038/nature12625

[b33] Whitehead R. H., Macrae F. A. St, John D. J. & Ma J. A colon cancer cell line (LIM1215) derived from a patient with inherited nonpolyposis colorectal cancer. J. Natl Cancer Inst. 74, 759–765 (1985). 3857372

[b34] Chen D. . TAp73 promotes cell survival upon genotoxic stress by inhibiting p53 activity. Oncotarget 5, 8107–8122 (2014). 25237903 10.18632/oncotarget.2440 PMC4226670

[b35] Wood K. C. . MicroSCALE screening reveals genetic modifiers of therapeutic response in melanoma. Sci. Signal. 5, rs4 (2012). 22589389 10.1126/scisignal.2002612 PMC3498828

