# Supplementary material for: Vertical suppression of the EGFR pathway prevents onset of resistance in colorectal cancers
Source: Nat Commun. 2015 Sep 22;6:8305. doi: 10.1038/ncomms9305 (PMC4595628; doi:10.1038/ncomms9305)
Supplement: Supplementary Information — Supplementary Figures 1-8, Supplementary Tables 1-4 and Supplementary Methods [file ncomms9305-s1.pdf]

Supplementary Figure 1

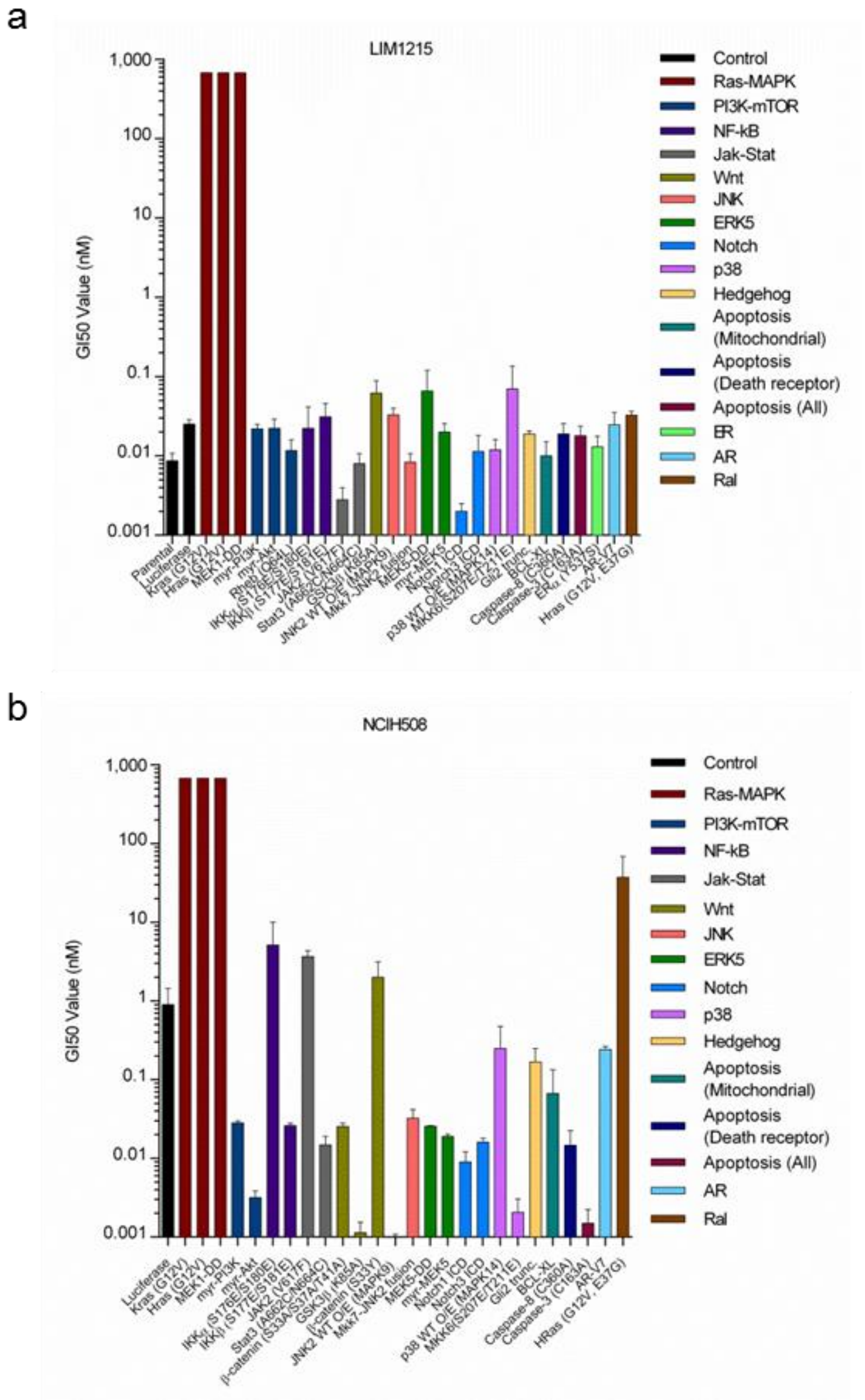

Supplementary Figure 1: To identify genes conferring resistance to anti EGFR antibodies LIM1215 (a) and NCIH508 (b) cells were infected with pathway specific cDNAs and treated with panitumumab. GI50 values are reported in logarithmic scale. Data are representative of three biological replicates and error bars represent standard deviantions

Supplementary Figure 2

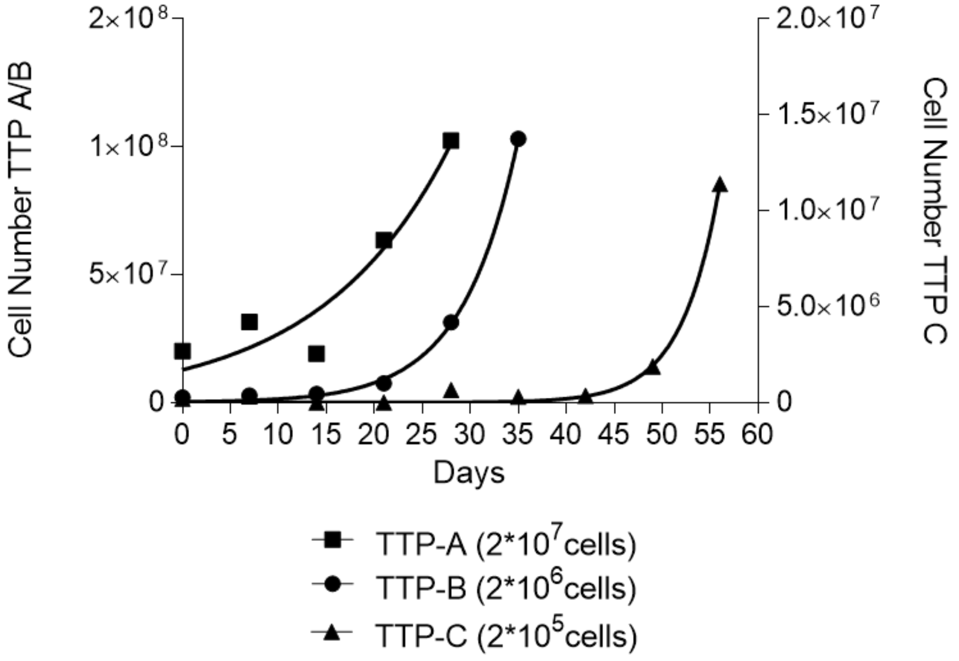

Supplementary Figure 2: Time of resistance depends on the initial cell input. LIM1215 cells were seeded at the indicated cell number and treated with the anti-EGFR monoclonal antibody cetuximab (340 nM). Data are representative of one biological replicate.

Supplementary Figure 3

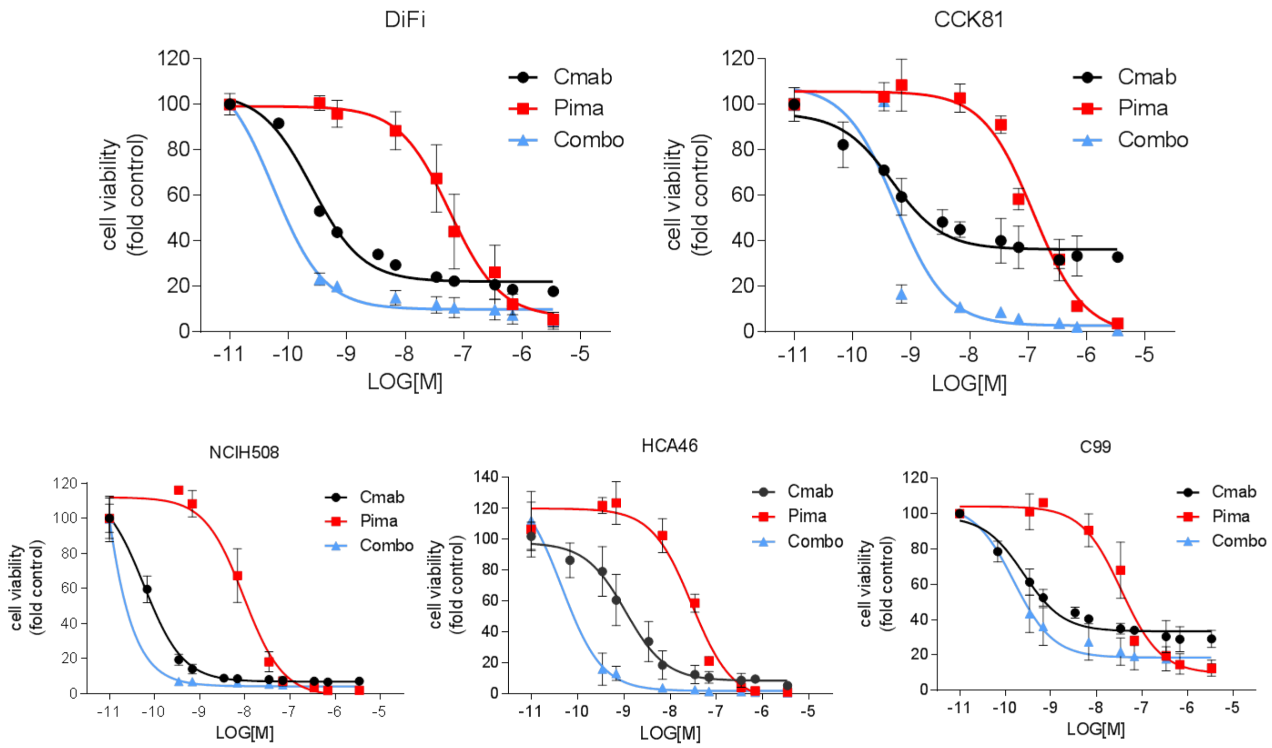

Supplementary Figure 3: DiFi, CCK81, NCIH508, HRA46 and C99 CRC cell lines were subjected to a dose-response curve with cetuximab, Pimasertib and the combination of a constant dose of Cetuximab (340 nM) and Pimasertib scale doses. The assay was performed in 6 days and curves are the average of three independent biological replicates.

Supplementary Figure 4

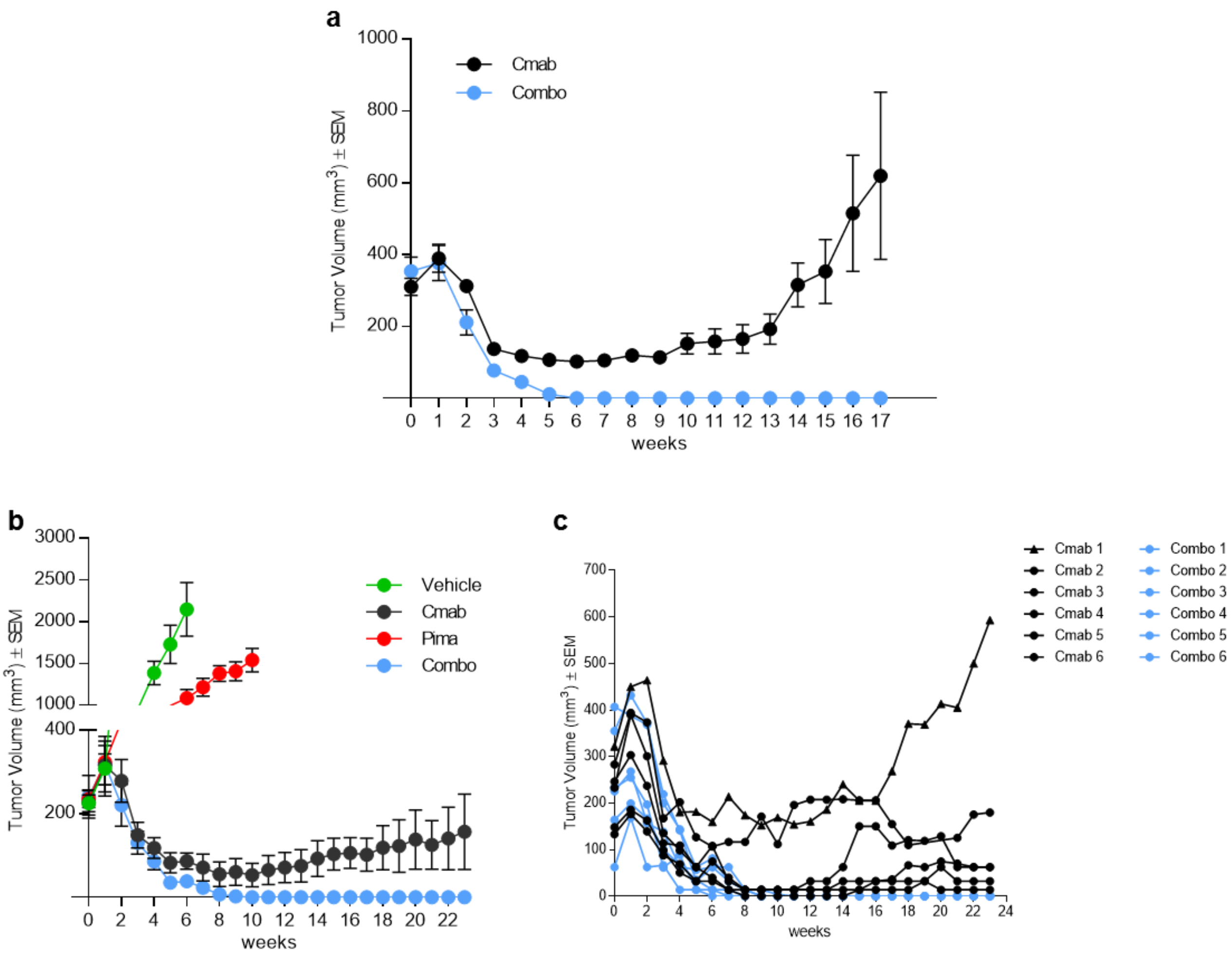

Supplementary Figure 4: (a) Technical replicate of the first PDX model (cetuximab 6 mice, combo 8 mice).Vehicle and pimasertib mono-treatment were not included as for ethical reasons Error bars represent SEM. (b) Second PDX model derived from a cetuximab sensitive patient: after randomization, mice were treated with vehicle alone, cetuximab, pimasertib or the combination (6 mice each) Error bars represent SEM. (c) Individual mice measurements showing a case (mouse cmab 1) of emerging resistance. In both cases treatment were initiated at week 2 (second PDX) or week 1 (second replicate of the first PDX) and not stopped.

Supplementary Figure 5

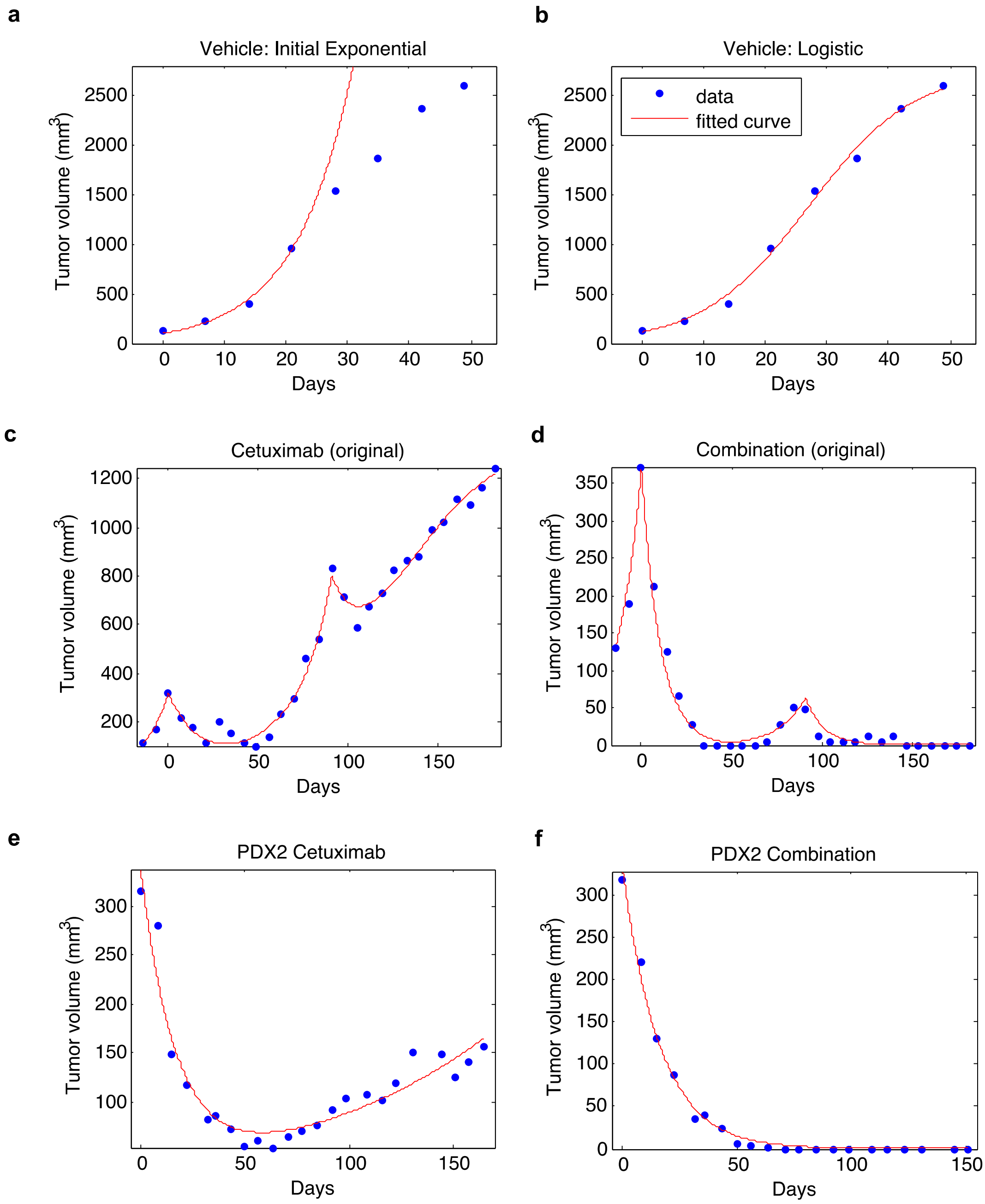

Supplementary Figure 5: Mathematical modeling of PDX experiments. (a-b) Fits of equations (1) (purely exponential initial growth) and (4) (logistic growth) from Supplementary Methods to the average tumor volume of untreated PDX1. (c) Fit of the treatment dynamics model that includes cessation and resuming of treatment, and includes both sensitive and resistant cells (see Supplementary Methods), to average tumor volume of PDX1 cetuximab treated original experiment. (d) Fit of the treatment dynamics model that includes cessation and resuming of treatment, and includes only sensitive cells (see Supplementary Methods), to average tumor volume of PDX1 combo treated original experiment. (e) Fit of model (1) to average tumor volume of cetuximab treated PDX2. (f) Fit of model (2) to average tumor volume of combo treated PDX2.

Supplementary Figure 6

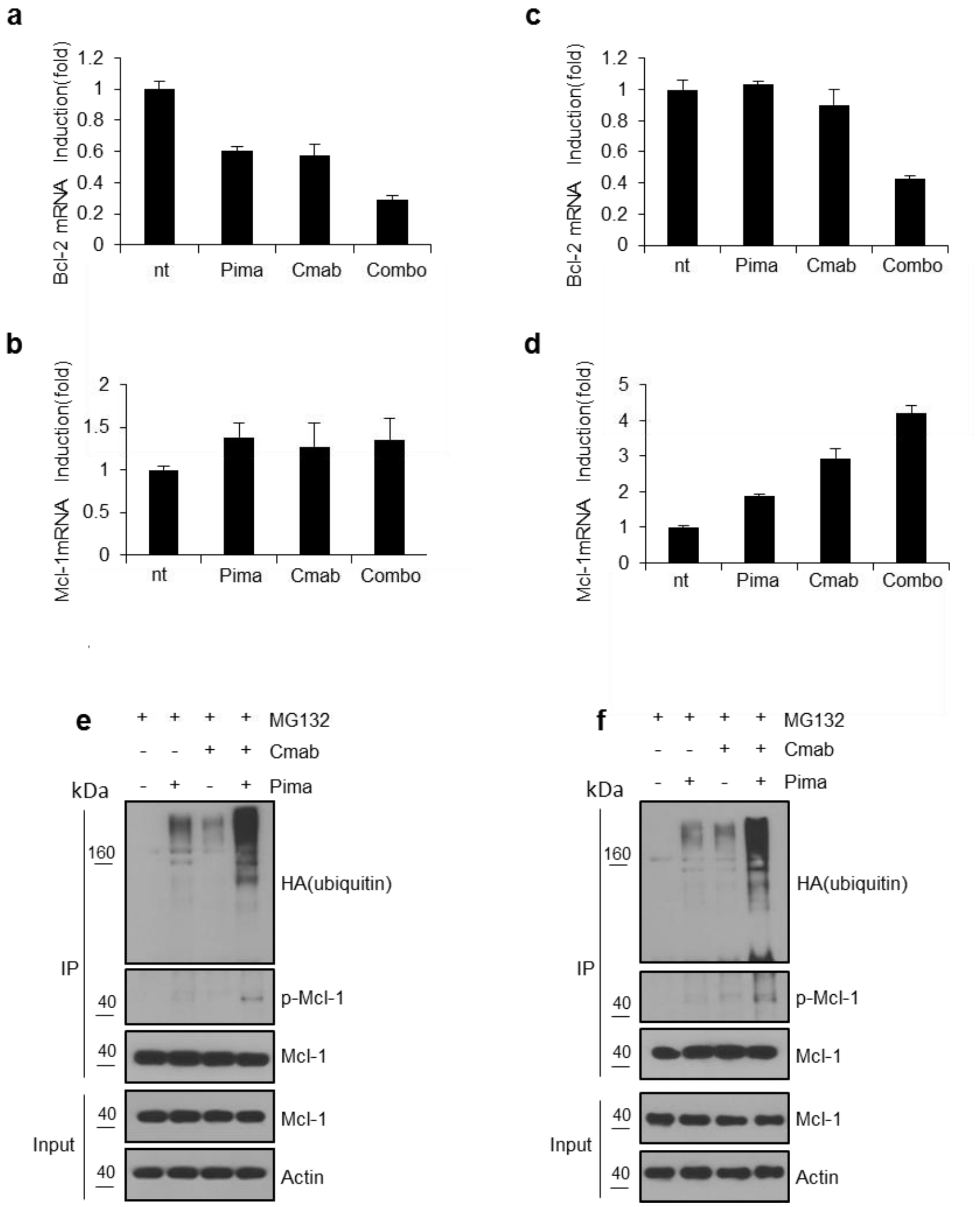

Supplementary Figure 6: EGFR and MEK concomitant inhibition modulates Bcl-2 and Mcl-1 expression and initiates apoptosis in CRC cell lines. (a-b) CCK81 and DiFi (c-d) cell lines were treated for 24 with cetuximab (340 nM), pimasertib (250 nM) or both. The levels of Bcl-2 and Mcl-1 mRNA were determined by Real-Time PCR analysis. (e) CCK81 and (f) DiFi cell lines were treated for 24 with cetuximab (340 nM), pimasertib (250 nM) or both with the proteasome inhibitor MG132. Western blotting was performed with the indicated antibodies. Actin was included as a loading control.

Supplementary Figure 7

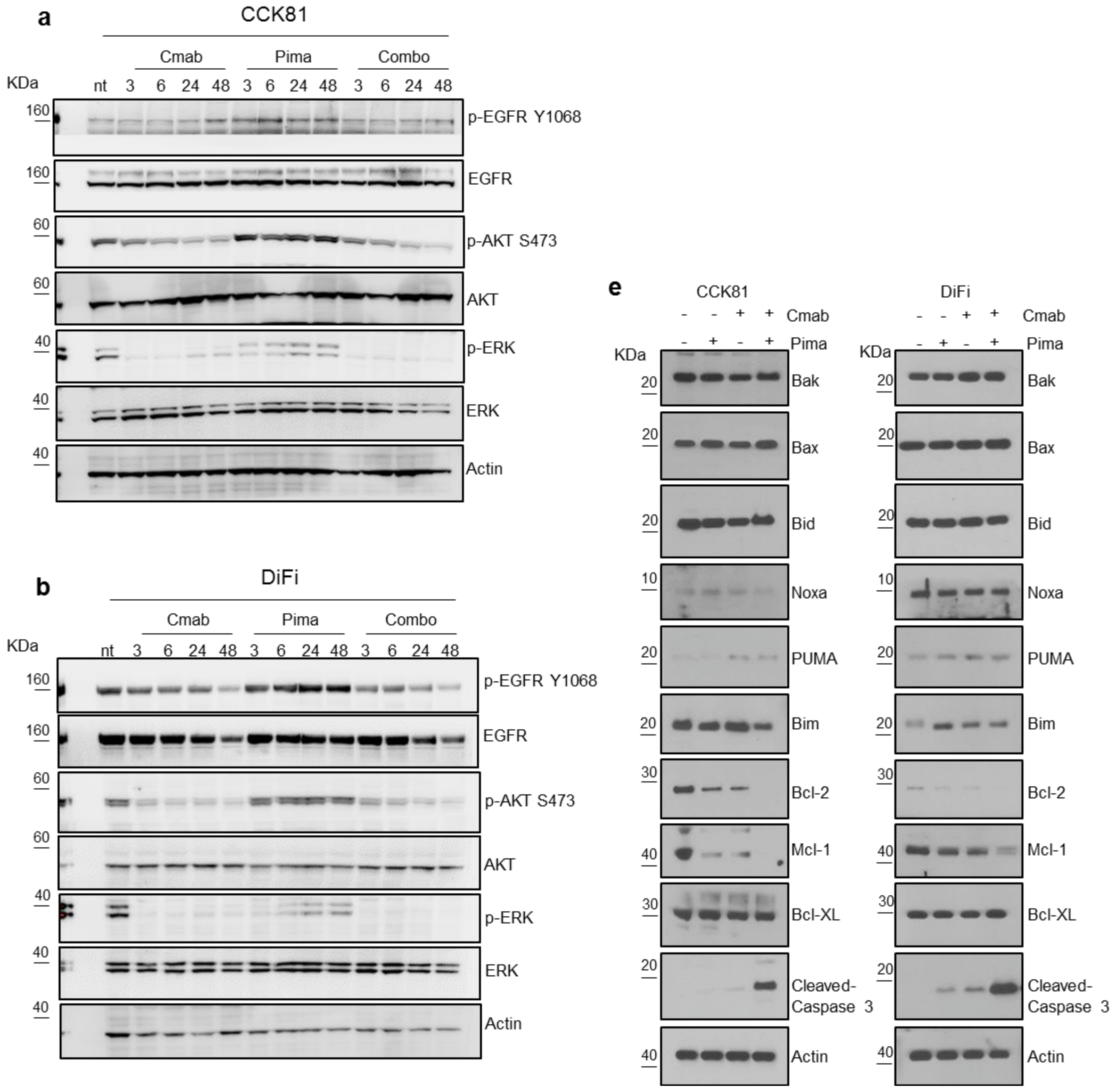

Supplementary Figure 7. Full lengths western blot of Figure 7. CCK81 (5a) and DiFi (5b) were treated with cetuximab (cmab, 340 nM), pimasertib (pima, 250 nM), or with the combo of the two drugs at the indicated time points, whole-cell extracts were subjected to Western blot analysis and compared to untreated cells with phospho-EGFR (Tyr 1068), total EGFR, total AKT and phospho-AKT (Ser 473), total ERK1/2 and phospho-ERK1/2 antibodies. Actin was included as a loading control. (5e) The indicated CRC cell lines were treated with cetuximab (cmab, 340 nM), pimasertib (pima, 250 nM), or with the two drugs for 48 hours. Whole-cell extracts were subjected to Western blot analysis and compared to untreated cells using BAK, Bax, Bid, NOXA, PUMA, Bim, Bcl-2, Mcl-1, Bcl-XL and active caspase-3 antibodies. Actin was included as a loading control.

Supplementary Figure 8

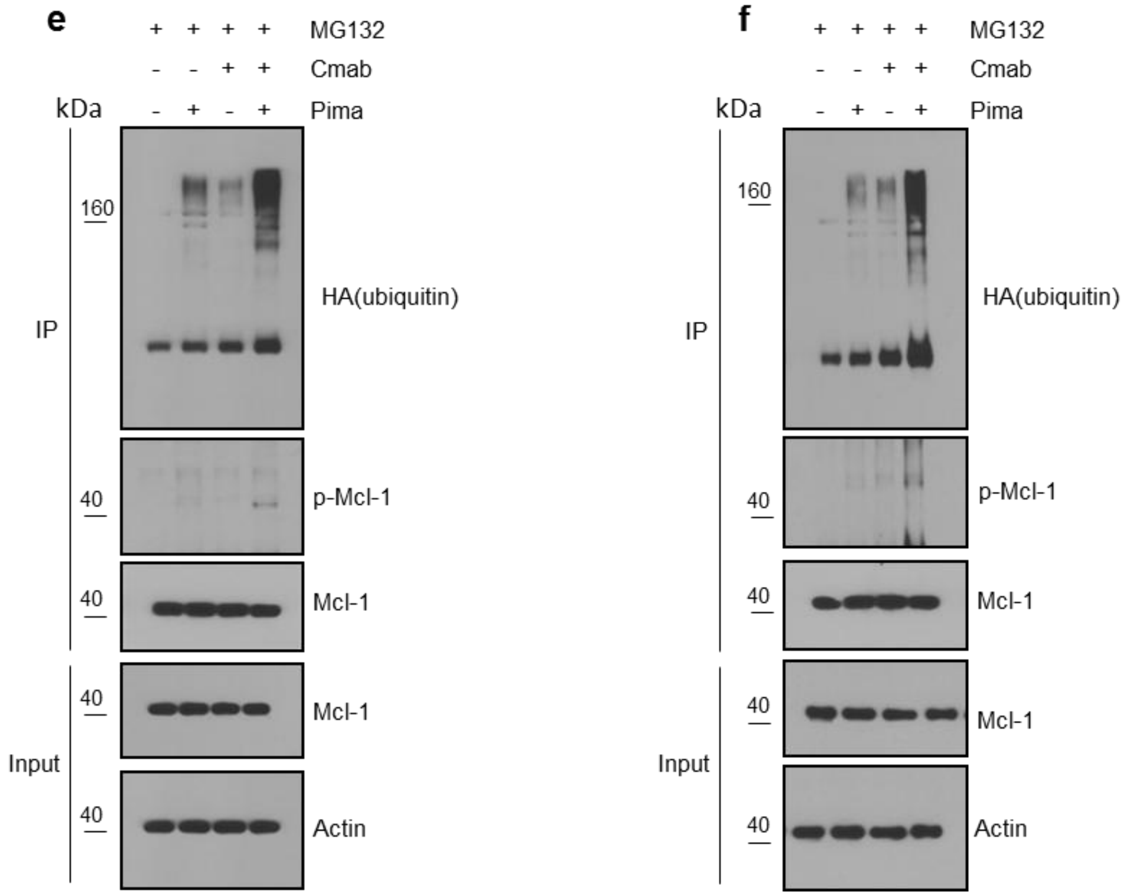

Supplementary Figure 8. Full lengths western blot of Supplementary Figure 7. (S7e) CCK81 and (S7f) DiFi cell lines were treated for 24 with cetuximab (340 nM), pimasertib (250 nM) or both with the proteasome inhibitor MG132. Western blotting was performed with the indicated antibodies. Actin was included as a loading control.

Supplementary Table 1

Pathway	ID	Construct	Construct type	Functionally validated	Functional Validation Method
**Ras-MAPK**	**A1**	Kras (G12V)	CA	Yes	Western (P-ERK)
	**A2**	Hras (G12V)	CA	Yes	Western (P-ERK)
	**A3**	MEK1 (S218D,S222D)	CA	Yes	Western (P-ERK)
**mTOR**	**B1**	myr-FLAG-PIK3CA	CA	Yes	Western (P-AKT)
	**B2**	myr-FLAG-AKT1	CA	Yes	Western (P-AKT, P-S6K1)
	**B3**	FLAG-Rheb (Q64L)	CA	Yes	Western (P-S6K1)
**NF-κB**	**C1**	IKKα (S176E,S180E)	CA	Yes	Reporter (NF-κB_Luc)
	**C2**	FLAG-IKKβ (S177E,S181E)	CA	Yes	Reporter (NF-κB_Luc)
**Jak/Stat**	**D1**	JAK2 (V617F)	CA	Yes	Reporter (Stat_Luc)
	**D2**	Stat3 (A662C,N664C,V667L)	CA	Yes	Reporter (Stat_Luc)
**Wnt/b-catenin**	**E2**	GSK3β (K85A)	DN	Yes	Reporter (TCF-LEF_Luc)
	**E3**	β-catenin (S33Y)	CA	Yes	Reporter (TCF-LEF_Luc)
**JNK**	**F1**	JNK2 WT O/E (MAPK9)	WT	No	Reporter (AP1_Luc)
	**F2**	Mkk7-JNK2 fusion	CA	Yes	Reporter (AP1_Luc)
**ERK5**	**G1**	MEK5 DD(S311D,T315D)	CA	No	Western (ERK5 laddering)
	**G2**	myr-FLAG -MEK5	CA	Yes	Western (ERK5 laddering)
**Notch**	**H1**	Notch1 intracellular domain	CA	Yes	Reporter (HES1_Luc)
	**H2**	Notch3 intracellular domain	CA	Yes	Reporter (HES1_Luc)
**p38**	**I1**	p38 WT O/E (MAPK14)	WT	Yes	Western (P-p38)
	**I2**	FLAG-MKK6 (S207E,T211E)	CA	Yes	Western (P-p38)
**Hedgehog**	**J1**	Gli2 truncation	CA	Yes	Reporter (Gli_Luc)
**Mitochondrial**	**L2**	BCL-XL	WT	Yes	Western (cleaved caspase 9)
**apoptosis**					
**(intrisic pathway)**					
**Death receptor**	**M1**	Caspase-8 (C360A)	DN	Yes	Western (cleaved caspase 8)
**apoptosis**					
**(extrisic pathway)**					
**All apoptosis**	**N1**	Caspase-3 (C163A)	DN	Yes	Western (cleaved caspase 3/7)
**Estrogen receptor**	**O1**	ERα (Y537S mutant)	CA	Yes	Reporter (ERE_Luc)
**Androgen receptor**	**P1**	AR-V7	CA	Yes	Western (ARE_Luc)
**Ral**	**S1**	HRas (G12V, E37G)	CA	Not tested	
**CONTROLS**	**X2**	Luciferase	Control	N/A	

List of cDNAs engineered to constitutively activate or inhibit the indicated signaling nodes[1]. WT = wild-type; CA = constitutively active; DN = dominant negative

Supplementary Table 2

Experiment	Parameter	Value	95% CI
Original	a	298.5	(234.3, 362.7)
	b	-0.06098	(-0.1018, -0.02013)
	c	21.42	(-15.02, 57.86)
	d	0.03607	(0.007102, 0.06503)
	e	0.02752	(0.01701, 0.03804)
	f	0.07827	(0.04058, 0.116)
	K	1368	(1070, 1666)
Replicate	a	417.2	(365, 469.5)
	b	-0.06169	(-0.07708, -0.0463)
	c	8.762	(2.754, 14.77)
	d	0.03843	(0.03137, 0.04549)

Best-fit parameters for the average tumor volume treated with cetuximab.

Supplementary Table 3

Experiment	Parameter	Value	95% CI
Original	a	368.1	(342.2, 394)
	b	-0.09696	(-0.107, -0.08687)
	f	0.07134	(0.0599, 0.08277)
Replicate	a	382.7	(367.2, 398.3)
	b	-0.09594	(-0.1036, -0.08832)

Best-fit parameters for the average tumor volume treated with combination.

Supplementary Table 4

Treatment	Parameter	Value	95% CI
Cetuximab	a	304.3	(267.7, 340.8)
	b	-0.05899	(-0.07456, -0.04343)
	c	33.84	(18.76, 48.91)
	d	0.009589	(0.006321, 0.01286)
Combination	a	328.8	(316.4, 341.2)
	b	-0.06111	(-0.06504, -0.05717)

Best-fit parameters for the average tumor volume of PDX 2 treated with cetuximab and combination.

Supplementary Methods:
Mathematical modeling of PDX experiments

PDX 1

Vehicle

In Supplementary Fig. 6a,b we plot the mean volume of the four untreated tumors. Initial growth (before tumor volume reaches ~ 1000 mm^3) of an untreated tumor can be well described by an exponential function

$$V(t) = a \exp(bt), \tag{1}$$

where V is tumor volume, t is time measured in days, a is the tumor volume at time $t = 0$ and b is the (fixed) growth rate of the tumor (Supplementary Fig. 6a). This exponential growth can equivalently be described using the differential equation

$$V' = bV. \tag{2}$$

Fitting formula (1) to the first four data points we obtain that the (initial) exponential growth rate of the untreated tumor is $b = 0.11$ $(95\%$ CI, $0.07 - 0.15)$ per day ($R^2 = 0.99$).

As the tumor size reaches ~ 1000 mm^3, tumor growth slows down, presumably due to spatial and nutrient limitations, and can be described by a logistic function

$$V' = bV\left(1 - \frac{V}{K}\right), \tag{3}$$

where b is the initial exponential growth rate and K is the carrying capacity of the tumor. The logistic function is explicitly given by

$$V(t) = \frac{Ka\exp(bt)}{K + a\left(\exp(bt) - 1\right)}. \tag{4}$$

Fitting formula (4) to the tumor volume data (Supplementary Fig. 6b) we obtain $b = 0.12$ $(95\%$ CI, $0.09 - 0.15)$ per day and $K = 2764$ $(95\%$ CI, $2430 - 3099)$ mm^3 ($R^2 = 0.995$).

Cetuximab (original experiment)

We plot the mean volume of four original tumors treated with cetuximab in Supplementary Fig. 6c. Initial treatment was started at day 0 and stopped after 42 days. However, the half-life of cetuximab is approximately seven days and we therefore assumed that effective treatment continued one week after delivery of drugs was stopped, i.e. until $t_1 = 49$. At day $t_2 = 91$ treatment was resumed and lasted until the end of the experiment. To model the

behavior of tumors under treatment, we will assume that at day 0 tumors contain a mix of sensitive and resistant cells. When treatment is on (day 0 to 49), we will describe the total tumor volume by

$$V(t) = a\exp(bt) + c\exp(dt). \tag{5}$$

Here a is the volume of sensitive cells in the tumor at time 0 and $b < 0$ is their (fixed) net growth rate during treatment. c is the volume of resistant cells at day 0 and $d > 0$ is their (fixed) growth rate during treatment. In other words, we assume that during treatment sensitive cells decline exponentially with rate b while resistant cells grow exponentially with rate d.

When treatment is off, prior to day 0 and between t_1 and t_2, the net growth rate of sensitive cells is $f > 0$, while the net growth rate of resistant cells is $e > 0$. Thus, for $t < 0$, tumor volume is given by

$$V(t) = a\exp(ft) + c\exp(et), \tag{6}$$

and for $t_1 < t < t_2$, tumor volume is given by

$$V(t) = a\exp(bt_1)\exp(f(t-t_1)) + c\exp(dt_1)\exp(e(t-t_1)). \tag{7}$$

After day $t_2 = 91$, when treatment is resumed, tumor volumes are in the vicinity of 1000 mm^3 and higher, so fitting them assuming pure exponential growth of resistant cells in unsuitable. For this reason, we will fit the growth of resistant cells using a logistic function described by the following growth law:

$$R' = dR\left(1 - \frac{R+S}{K}\right), \tag{8}$$

where R is the volume of resistant cells, and S is the volume of sensitive cells. Parameter K denotes the carrying capacity of the tumor. As during the initial treatment, sensitive cells behave according to

$$S' = bS, \tag{9}$$

where $b < 0$. In other words, they decline exponentially during treatment with rate b.

The model is an excellent fit to the data ($R^2 = 0.99$, Supplementary Fig. 6c). We show the best fit coefficients and their 95% confidence bounds for the average tumor volume during cetuximab treatment in Supplementary Table 2. According to the fitting results, the volume of sensitive cells at the start of treatment was $a = 298$ mm^3, and the volume of resistant cells was 21 mm^3. Thus $\sim 6.7\%$ of tumor cells present at the start of treatment were resistant to cetuximab. Growth rate of sensitive cells during treatment is $b = -0.06$ and their growth rate in the absence of treatment is $f = 0.08$ per day. Growth rate of resistant cells during treatment is $d = 0.04$ and in the absence of treatment, $e = 0.03$ per day. As the 95$\%$ confidence intervals for growth rates of sensitive and resistant cells in the absence of treatment, f and e, are completely non-overlapping, we can conclude that resistance is costly prior to treatment.

Finally, although the predicted volume of resistant cells is high, the confidence interval for resistant cells is wide and includes 0, so we cannot completely rule out that resistance was not present at the start of treatment. The width of the confidence intervals is likely affected by the large number of different parameters that are being fitted and the complicated setting of the experiment. For that reason we have repeated this experiment in a setting in which treatment is continuous.

Cetuximab (replicate experiment)

We have repeated cetuximab experiments with the same PDX, this time with no stopping of treatment. Tumor volumes are shown in Fig. 4a. This allows us to fit a single simple model of tumor volume to the data:

$$V(t) = a\exp(bt) + c\exp(dt), \tag{10}$$

with parameters a, b, c and d as defined in the previous section. The model fit the data well ($R^2 = 0.97$, Fig. 4a). Best-fit parameters for the average tumor volume are shown in Supplementary Table 2. The net growth rates of sensitive and resistant cells during treatment (b and d) are in excellent agreement with parameters estimated from the previous experiment (Supplementary Tables 5). The predicted volumes of sensitive and resistant cells at the start of treatment are 417 and 8.8 mm^3. Thus $\approx 2\%$ of cells present at the start of treatment were resistant to cetuximab. The 95% CI for the volume of resistant cells is (2.754, 14.77) mm^2, and does not include 0, so we can conclude that resistant cells were present at the start of treatment.

Combination (original experiment)

Combination of cetuximab and pimasertib was applied to the same PDX in a manner similar to original treatment with cetuximab. Combination treatment was initiated at day 0 and lasted until day $t_1 = 49$. Treatment was resumed on day $t_2 = 91$ and lasted until the end of experiment (day 182). Average tumor volume of the four mice treated with combination is shown in Supplementary Fig. 6d.

During initial treatment, tumor shrank to below detection (Supplementary Fig. 6d). After treatment is stopped (day 49), the tumor resumes growth. However, retreatment leads to renewed tumor shrinkage and tumor remains undetectable until the end of experiment. Thus we conclude that the tumor growth during the time without treatment was due to the growth of cells sensitive to combination therapy. We see no evidence of combination therapy resistant cells in tumor volume data, and thus we fit a model that contains only sensitive cells (Supplementary Fig. 6d). The model assumes that sensitive cells decline with rate $b < 0$ during treatment, and grow with rate $f > 0$ in the absence of treatment. Volume of sensitive cells at the start of therapy (day 0) is a. Best-fit parameters are shown in Supplementary Table 3 ($R^2 = 0.98$).

Combination (replicate experiment)

We have repeated the same experiment with continuous treatment (shown in Fig. 4b). As there is no sign of regrowth of resistant cells, we again fit a model that contains only sensitive cells:

$$V(t) = a\exp(bt). \tag{11}$$

The model was an excellent fit to the data ($R^2 = 0.99$). Best fit parameters are shown in Supplementary Table 3 and they are in great agreement with estimates from the previous section.

PDX 2

Cetuximab

We fit the same model as in the PDX 1 replicate experiment:

$$V(t) = a\exp(bt) + c\exp(dt). \tag{12}$$

As before, a is the volume of sensitive cells in the tumor at time 0 and $b < 0$ is their (fixed) net growth rate during treatment. c is the volume of resistant cells at day 0 and $d > 0$ is their (fixed) growth rate during treatment. Best-fit parameters are shown in Supplementary Table 4, and the fit is shown in Supplementary Fig. 6e ($R^2 = 0.93$).

Combination

We fit the same model as in the PDX 1 replicate experiment with combination therapy:

$$V(t) = a\exp(bt). \tag{13}$$

The model was again an excellent fit to the data ($R^2 = 0.995$, Supplementary Fig. 6f). Best fit parameters are shown in Supplementary Table 4.
